# Value of a catch-up HPV test in women aged 65 and above: A Danish population-based nonrandomized intervention study

**DOI:** 10.1371/journal.pmed.1004253

**Published:** 2023-07-06

**Authors:** Mette Tranberg, Lone Kjeld Petersen, Anne Hammer, Miriam Elfström, Jan Blaakær, Susanne Fogh Jørgensen, Mary Holten Bennetsen, Jørgen Skov Jensen, Berit Andersen

**Affiliations:** 1 University Research Clinic for Cancer Screening, Department of Public Health Programmes, Randers Regional Hospital, Randers, Denmark; 2 Department of Obstetrics and Gynecology, Odense University Hospital, Odense, Denmark; 3 OPEN, Department of Clinical Medicine, Southern University of Denmark, Odense, Denmark; 4 Department of Clinical Medicine, Aarhus University, Aarhus, Denmark; 5 Department of Obstetrics and Gynecology, Gødstrup Hospital, Herning, Denmark; 6 Center for Cervical Cancer Prevention, Karolinska University Hospital, Stockholm, Sweden; 7 Regional Cancer Center of Stockholm-Gotland, Stockholm, Sweden; 8 Department of Clinical Research, University of Southern Denmark, Odense, Denmark; 9 Department of Pathology, Randers Regional Hospital, Randers, Denmark; 10 Research Unit for Reproductive Microbiology, Statens Serum Institut, Copenhagen, Denmark

## Abstract

**Background:**

High-risk human papillomavirus (HPV) test is replacing cytology as the primary cervical cancer screening test due to superior sensitivity, but in most countries women ≥65 years have never had an HPV test despite they account for around 50% of cervical cancer deaths. We explored the effect of a catch-up HPV test among 65- to 69-year-old women without previous record of HPV-based screening.

**Methods and findings:**

This population-based nonrandomized intervention study (quasi-experimental design) included Danish women aged 65 to 69 with no record of cervical cancer screening in the last ≥5.5 years and no HPV-exit test at age 60 to 64 at the time of study inclusion. Eligible women residing in the Central Denmark Region were invited for HPV screening either by attending clinician-based sampling or requesting a vaginal self-sampling kit (intervention group, *n* = 11,192). Women residing in the remaining four Danish regions received standard care which was the opportunity to have a cervical cytology collected for whatever reason (reference group, *n* = 33,387). Main outcome measures were detection of cervical intraepithelial neoplasia (CIN) grade 2 or worse (CIN2+) per 1,000 women eligible for the screening offer and the benefit–harm ratio of the intervention and standard practice measured as the number of colposcopies needed to detect one CIN2+ case. The minimum follow-up time was 13 months for all tested women (range: 13 to 25 months). In the intervention group, 6,965 (62.2%) were screened within 12 months from the date of study inclusion and 743 (2.2%) women had a cervical cytology collected in the reference group. The CIN2+ detection was significantly higher in the intervention group (3.9, 95% confidence interval (CI): [2.9, 5.3]; *p* < 0.001; *n* = 44/11,192) as compared to the reference group (0.3, 95% CI: [0.2, 0.6]; *n* = 11/33,387). For the benefit–harm ratio, 11.6 (95% CI: [8.5, 15.8]; *p* = 0.69; *n* = 511/44) colposcopies were performed to detect one CIN2+ in the intervention group as compared to 10.1 (95% CI: [5.4, 18.8]; *n* = 111/11) colposcopies in the reference group. The study design entails a risk of confounding due to the lack of randomization.

**Conclusions:**

The higher CIN2+ detection per 1,000 eligible women in the intervention group supports that a catch-up HPV test could potentially improve cervical cancer prevention in older women. This study informs the current scientific debate as to whether women aged 65 and above should be offered a catch-up HPV test if they never had an HPV test.

**Trial registration:**

ClinicalTrials.gov NCT04114968.

## Abstract 2

In a population based cohort study, Mette Tranberg and colleagues investigate the value of catch-up HPV testing in more than 45,000 eligible women from Denmark aged over 65 years.

## Author summary

**Why was this study done?:**

- Recommendations on cervical cancer screening at older ages are mainly based on expert opinions and modeling studies rather than empirical evidence.
- High-risk human papillomavirus (HPV) test is replacing cytology as the primary cervical cancer screening test due to superior sensitivity, but in most countries women over 65 years have never had an HPV test and controversy remains on whether and how these women should be offered a catch-up HPV test to prevent cancer at older ages.
- Observational studies have shown only that women who have been insufficiently screened at age 50 to 64 could benefit from continued screening after the age of 65 but evidence on the optimal screening tool to reach insufficiently screened women above 65 is missing.

**What did the researchers do and find?:**

- This population-based study including a total of 44,579 women showed that a catch-up HPV screening intervention, with the opportunity to choose between clinician-based sampling and vaginal self-sampling, was associated with significantly higher detection of cervical intraepithelial neoplasia (CIN) grade 2 or worse (CIN2+) per 1,000 eligible women as compared to women not offered screening.
- This study demonstrated that women who have been insufficiently screened at age 50 to 64 (≤1 cytology sample) had higher HPV prevalence and tended to have more CIN2+ lesions diagnosed as compared to sufficiently screened women (≥2 cytology samples).
- Insufficiently screened women were more likely to undergo vaginal self-sampling as compared to sufficiently screened women, which supports that this screening modality might be ideal to identify older women at risk of cervical cancer.

**What do these findings mean?:**

- Evidence presented in this study suggests that a catch-up HPV screening intervention could potentially improve cervical cancer prevention in women aged ≥65. Going forward, longer follow-up of the screened women will show if the intervention results in fewer cervical cancer cases and deaths.
- Policy makers should consider whether HPV catch-up screening interventions should be differentiated depending on previous screening participation and results to reach an appropriate cost-benefit balance.

## Introduction

The relatively high incidence and mortality of cervical cancer in women above the current screening age have raised concern in countries with well-established cervical cancer screening programs [1–4]. With an increasing life expectancy and decreasing hysterectomy rates [5,6], the incidence of cervical cancer in women aged 65 and above might be expected to rise even more than already seen [4]. As more well-screened birth cohorts age through the screening program, future data will show whether the incidence in older ages will decrease. While lack of evidence remains on the optimal upper age limit for screening, and most screening recommendations therefore are based on expert opinions [7,8] and modeling studies [9], observational studies have reported that women who had been insufficiently screened or had abnormal cytological screening between the ages of 50 and 64 could benefit from continued screening after the age of 65 [10,11]. However, almost 40% of cervical cancers diagnosed at age ≥65 in the United Kingdom occur among sufficiently screened women who exited the program with normal cytology results [12], which may suggest a reduced sensitivity of cytology-based screening at older ages [13]. Several countries are currently transitioning from cytology-based screening to the more sensitive high-risk human papillomavirus (HPV)-based screening method [14,15]. To detect disease among older women that may have been missed by cytology, it has been suggested to offer a “catch-up” HPV test to women over 65 who have never had an HPV test [12]. In Denmark, women aged 69 and above were offered a one-time HPV screening test at their general practitioner (GP) in 2017 [16], while one Swedish county, as part of switching to HPV-based screening, offered an HPV test to a small cohort of women aged 70 in 2016 [17]. The effectiveness of an HPV catch-up screening intervention will depend on the uptake and, in particular, on its ability to reach insufficiently screened women [12]. The urgency to reach these women is emphasized by data from the UK reporting that women who have not been screened since the age of 50 are almost six times more likely to develop cervical cancer at age 65 and older than those sufficiently screened [11]. Common barriers towards clinician-based cervical cancer screening include discomfort and pain during the gynecological examination [18]. To overcome these barriers, home-based vaginal self-sampling for HPV testing could be an attractive [19], accurate [20], and cost-effective screening alternative for women older than 65 [21]. Yet, the benefit of offering self-sampling to older insufficiently screened women remains unknown. From a clinical perspective, colposcopy and biopsy are challenging in older women as the majority will have a nonvisible transformation zone, resulting in lengthy follow-up with repeated cervical sampling to obtain representative biopsies and increased risk of missing disease [22,23]. Besides, referral for colposcopy and overdiagnosis (i.e., detection of transient HPV infections) are known harms of screening as they can lead to psychological distress and anxiety [24,25]. Therefore, it must be carefully evaluated whether the potential harms of an HPV catch-up test to women aged 65 and above are outweighed by the potential benefits of reaching insufficiently screened women and increased detection of cervical intraepithelial neoplasia (CIN) grade 2 or worse (CIN2+).

We evaluated whether a catch-up HPV screening intervention, with the opportunity to choose between vaginal self-sampling and clinician-based sampling, was associated with high screening uptake and higher CIN2+ detection as compared to women not offered HPV screening. Secondly, we evaluated if insufficiently screened women were more likely to undergo vaginal self-sampling than sufficiently screened women. Finally, we estimated the benefit–harm ratio of the intervention as number of colposcopies needed to detect one CIN2+ case.

## Methods

This study was reported using the Transparent Reporting of Evaluations with Nonrandomized Designs (TREND) statement checklist (S1 Text) [26].

### Ethics statement

The study was listed at the record of processing activities for research projects in the Central Denmark Region (j.no: 1-16-02-158-18). The study was approved by the Danish Patient Safety Authority (j. no: 3-3013-2634/1). The study has been submitted to the Ethical Committee in the Central Denmark Region. The Committee decided that this study was not notifiable to the Committee (j. no.: 73/2018) and informed consent was therefore not required.

### Study setting

Denmark has had an organized free-of-charge cervical cytology-based screening program targeting women aged 23 to 59 years since the late 1990s [27]. In 2007, the upper age limit was extended to age 64 with five-year screening intervals in women aged 50 years and older, and since 2012 women aged 60 to 64 have been offered an HPV-DNA exit test but nationwide implementation was first achieved in 2014. HPV–negative women exit the program without consideration of their previous screening history [27]. The Danish program is based on an integrated call-recall invitation module using data from the nationwide Danish Pathology Data Bank (DPDB) that hold records on all pathology specimens [28]. The present study was carried out at the Department of Public Health Programmes, Randers Regional Hospital, which is responsible for running the cervical cancer screening program in the Central Denmark Region (CDR). The region covers approximately one-fifth of the Danish population (1.2 million inhabitants), including 345,000 women in the target population for cervical cancer screening [29].

### Design and study population

In this nationwide, population-based nonrandomized intervention study (i.e., a quasi-experimental design), 65- to 69-year-old women were eligible for inclusion if they, between 9 April and 8 May 2019: (1) were residing in Denmark; (2) had no record of a cervical cytology sample or screening invitation in the preceding 5.5 years or more; (3) no record of an HPV-exit test at age 60 to 64; (4) were not registered in the invitation module as having actively opted out of the screening program; and (5) had no record of total hysterectomy or cervical amputation. Women fitting the inclusion criteria were ascertained using data from the invitation module, DPDB [28], and the Danish National Patient Registry [30]. Eligible women residing in the CDR at the date of inclusion were allocated to the intervention group, whereas women residing in the remaining four Danish regions were allocated to the reference group. Women were excluded if they died, emigrated, and/or had migrated between the intervention region and reference regions between the date of inclusion and end of follow-up (i.e., 1 year later). This present study was conducted alongside a population-based intervention study evaluating the effectiveness of expanding the upper screening age from 65 to 69 years [31]. The present study’s intervention, follow-up protocol, main outcomes, statistical analyses, and identification of reference group were conducted in line with this published study protocol [31].

### Data sources

Data on hysterectomy or cervical amputation performed from 1 January 1999 to 8 May 2019 was collected from the Danish National Patient Registry (S2 Text). Information on death, emigration, and migration was retrieved from the Danish Civil Registration System [32]. The DPDB has been considered complete since the mid-2000s and was in this study used to retrieve information on the dates and HPV-result of the clinician-collected cervical cytology samples and vaginal self-samples as well as results of any triage and/or follow-up test [28]. Cervical histology results were divided into <CIN2 (normal including inflammation and nonspecific reactive features and CIN1) and CIN2+ (CIN2, CIN3, AIS (adenocarcinoma in situ), unclassifiable CIN (i.e., the full height of the epithelium is not discernible), and cancer). Histological diagnosis was based on the result of cervical biopsies, endocervical curettage (ECC), or large loop excision of the transformation zone (LLETZ). The most severe diagnosis during follow-up was used. Follow-up concluded on 11 June 2021 so that the minimum follow-up time after the index test was at least 13 months for all women tested (range 13 to 25 months). To account for potential confounding of the study outcomes, data on women’s screening history were retrieved. Women were categorized as insufficiently screened if they had ≤1 cervical cytology sample and as sufficiently screened if ≥2 cervical samples at age 50 to 64 were recorded in the DPDB [28]. This study used linkage of data at an individual level, which is possible owing to the unique Civil Personal Registration (CPR) number, which is assigned to all residents at birth or upon immigration [32].

### Intervention group

The intervention group was invited to HPV-based cervical cancer screening and targeted women could choose to book an appointment for a clinician-collected cervical sample at their general practitioner (GP) or order a self-sampling kit. The kit included a dry brush device (Evalyn Brush, Rovers Medical Devices, B.V, Oss, the Netherlands), written and picture-based user instructions, and a pre-stamped return envelope [31]. All information was in Danish. Non-participants received reminder letters after 3 and 6 months.

### Sample collection, processing, and follow-up

In the intervention group, all clinician-collected cervical samples and self-samples were stored in 10 mL SurePath medium (BD Diagnostics, Burlington, North Carolina, United States of America) prior to HPV testing at Randers Pathology Department. HPV DNA testing was performed using the COBAS 4800 assay (Roche Diagnostics, Switzerland), which provides results for HPV16, HPV18 and pooled detection of 12 other HPV types (HPV31, 33, 35, 39, 45, 51, 52, 56, 58, 59, 66, and 68) [33]. Women with HPV–positive cervical samples were subjected to reflex cytology and the Bethesda system 2014 was used for cytopathological classification.

### Follow-up algorithm

Test results and follow-up recommendations were sent to the woman with a copy to her GP. Details of the follow-up algorithms are described elsewhere [31]. In accordance with Danish guidelines [34], women with HPV–negative samples ended follow-up. Direct referral to colposcopy was recommended for women who were positive for HPV16 and/or HPV18, or were HPV–positive for other types and had atypical squamous cells of undetermined significance (ASC-US) or worse in their clinician-based sample. Women positive for HPV types other than HPV16/18 and normal cytology were recommended a follow-up cervical screening test after 12 months, which underwent HPV and cytology co-testing. Women were only referred for colposcopy if either test result was positive. Women with an HPV–positive self-sample were recommended cytology follow-up testing by their GP within 30 days to evaluate the need for referral to colposcopy, and this sample underwent HPV and cytology co-testing. For women referred for colposcopy, it was recommended to collect 4 cervical punch biopsies, irrespective of colposcopic findings or cytology result, which is in accordance with national guidelines [35]. Some women in the intervention group may have undergone a diagnostic LLETZ [22].

### Reference group

The reference group reflected usual practice which, for 65 to 69 years old women, was the opportunity to have an opportunistic clinician-collected cervical sample obtained at their GP or by a gynecologist at their own or the physicians initiative for whatever reason (e.g., postmenopausal bleeding). These women received no screening invitation but were assigned individual dates of eligibility for study inclusion allowing comparison between the groups.

### Outcomes and statistical analyses

The primary outcomes in the two groups were the proportion of tested women, the number of CIN2+ lesions detected per 1,000 women who were eligible for the intervention, reflecting an intention-to-treat analysis, and the benefit–harm ratio. CIN2+ was chosen as main clinical outcome since it is the treatable threshold for older women. The CIN2+ detection in the intervention group was also reported using the total number of HPV–positive women with histology as denominator and according to HPV genotype (HPV16/18 versus HPV other types). The proportion of tested women was calculated as the number of cervical cytology samples or vaginal self-samples (defined as index test) registered within 12 months from the date of eligibility for study inclusion divided by the number of women eligible for the screening offer. This outcome was tabulated by screening modality, age group, and screening history. We evaluated the benefit–harm ratio of the intervention and standard practice by estimating the number of colposcopies required to detect a single case of CIN2+/ CIN3+ (CIN3/AIS and cancer) as previously described [16]. Among tested women, a record of cervical biopsies, ECC or LEETZ registered on the same day during the 25-month study period was used as a proxy for having a colposcopy performed. Other outcomes considered the (1) HPV prevalence, calculated as the number of HPV–positive samples divided by the number of tested women and if multiple HPV infections were detected, we used a hierarchical classification, assuming HPV 16 and/or 18 to be the causal genotypes [36]; and (2) proportion of HPV–positive self-samplers attending the recommend cytology follow-up testing at the GP within 180 days. Proportions were calculated based on the exact binomial distribution and reported with 95% confidence intervals (CI). Differences between proportions were tested with two-sample test. *P*-values <0.05 were considered statistically significant. Statistical analyses were performed using STATA version 16. Other outcomes in the intervention group such as sociodemographic characteristics between participants and non-participants as well as triage and follow-up test results in participating women will be reported elsewhere.

## Results

### Study population

**Fig 1:**
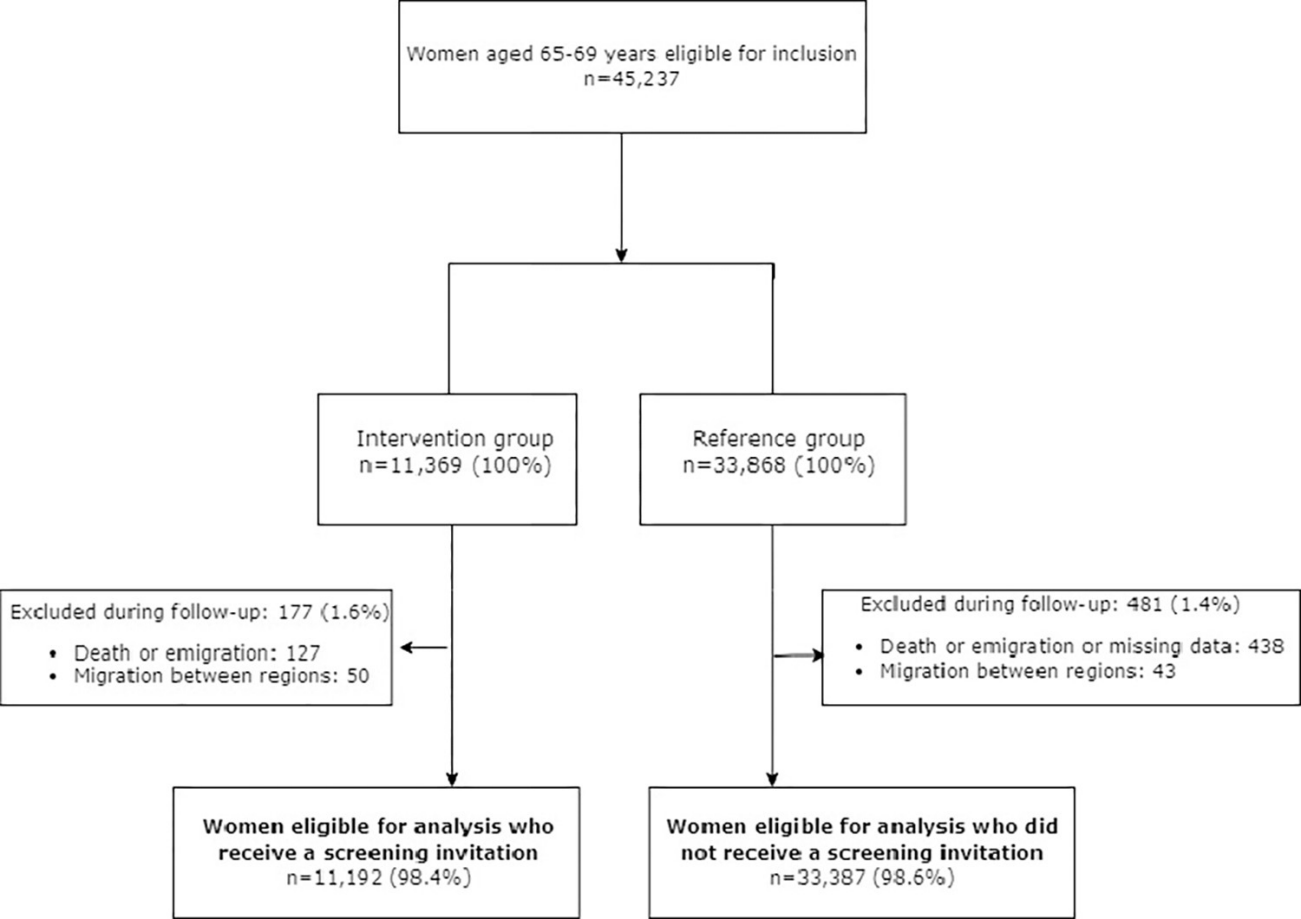
Flowchart of the study design.

From a total of 45,237 women eligible, 11,369 and 33,868 were allocated to the intervention and reference groups, respectively (Fig 1). After exclusions during follow-up, 11,192 (98.4%) women in the intervention group and 33,387 women (98.6%) in the reference group were included for analysis (Fig 1). The median age was 68.4 and 68.5 years in the intervention and reference groups, respectively (Table 1). In both groups, a majority of women (>76%) had been sufficiently screened at age 50 to 64.

**Table 1:** Characteristics of the study population.

	Intervention group *n* = 11,192	Reference group *n* = 33,387	*p*-value*
**Median age (years) at inclusion**	68.4	68.5	
**Age (years) at inclusion**			
65	242 (2.1)	421 (1.3)	*p* < 0.001
66	1,550 (13.9)	3,658 (10.9)	
67	2,470 (22.1)	7,510 (22.5)	
68	3,412 (30.5)	10,464 (31.3)	
69	3,518 (31.4)	11,334 (34.0)	
**Screening at age 50–64**			
Insufficiently screened (≤1 cervical sample)	2,665 (23.8)	7,636 (22.9)	*p* = 0.04
Sufficiently screened (≥2 cervical samples)	8,527 (76.9)	25,751 (77.1)	

Data are provided as number (column percent).

* Chi square test.

### Proportion of tested women

In the intervention group, a total of 6,965 (62.2%, 95% CI: [61.3, 63.1%]) women were tested. Of those tested, more women underwent clinician-based sampling than vaginal self-sampling (71.1%, 95% CI: [70.0, 72.2%] versus 28.9%, 95% CI: [27.8, 30.0%], *p* < 0.001) (Table 2). Proportion of tested women varied from 63.1% (95% CI: [62.0, 64.2%]) at age 68 to 69 years to 60.9% (95% CI: [59.4, 62.3%]; *p* = 0.02) at age 65 to 67 years. Insufficiently screened women were more likely to undergo self-sampling as compared to sufficiently screened women (52.1%, 95% CI: [47.4, 56.9%] versus 27.3%, 95% CI: [26.2, 28.4%]; *p* < 0.001). In the reference group, a total of 743 women (2.2%, 95% CI: [2.1, 2.4%]) had a record of a cervical sample (Table 2), and most of these women underwent HPV testing (81%, 601/743) (Table 3).

**Table 2:** Tested women, by age and screening history.

	Intervention group				Reference group	
	Eligible women *N*	Total tested *n* (% of eligible)	Clinician-based sampling *n* (% of total tested)	Self-sampling *n* (% of total tested)	Eligible women *N*	Total tested* *n* (% of eligible)
	11,192	6,965 (62.2)	4,955 (71.1)	2,010 (28.9)	33,387	743 (2.2)
**Age groups (years)**						
65–67	4,262	2,594 (60.9)	1,831 (70.6)	763 (29.4)	11,589	312 (2.7)
68–69	6,930	4,371 (63.1)	3,124 (71.5)	1,247 (28.5)	21,798	431 (2.0)
**Screening at age 50–64**						
Insufficiently screened (≤1 cervical sample)	2,665	443 (16.6)	212 (47.9)	231 (52.1)	7,636	65 (0.9)
Sufficiently screened (≥2 cervical samples)	8,527	6,522 (76.5)	4,743 (72.7)	1,779 (27.3)	25,751	678 (2.6)

Data are provided as numbers (row percent).

*All clinician-based sampling.

**Table 3:** HPV test results.

	Intervention group								Reference group			
	Clinician-based sampling		Self-sampling		Total				Total			
Age groups (years)	Tested women	HPV-positive	Tested women	HPV-positive	Tested women	HPV-positive	HPV16/18% of HPV positive	HPV Other % of HPV positive	Tested^#^ women	HPV-positive	HPV16/18% of HPV positive	HPV Other% of HPV positive
65–67	1,831	121 (6.6)	763	68 (8.9)	2,594	189 (7.3)	49 (25.9)	140 (74.1)	312	8 (2.6)	3 (37.5)	5 (62.5)
68–69	3,124	173 (5.5)	1,247	93 (7.5)	4,371	266 (6.1)	67 (25.2)	199 (74.8)	431	22 (5.1)	11 (50.0)	11 (50.0)
**Total**	4,955	294 (5.9)	2,010	161 (8.0)	6,965	455 (6.5)	116 (25.5)	339 (74.5)	743	30 (4.0)	14 (46.7)	16 (53.3)
**Screening at age 50–64**												
Insufficiently screened (≤1 cervical sample)	212	26 (12.3)	231	25 (10.8)	443	51 (11.5)	8 (15.7)	43 (84.3)	65	4 (6.2)	3 (75.0)	1 (25.0)
Sufficiently screened (≥2 cervical samples)	4,743	268 (5.7)	1,779	136 (7.6)	6,522	404 (6.2)	108 (26.9)	296(73.3)	678	26 (3.8)	11 (42.3)	15 (57.7)

Data are provided as number (row percent).

^#^A combination of cytology and HPV testing with a total of 601 women (81%) who underwent HPV testing.

HPV, human papillomavirus. HPV positive: HPV16 and/or HPV18 and/or 12 other high risk HPV types (HPV31, 33, 35, 39, 45, 51, 52, 56, 58, 59, 66 and 68).

### HPV prevalence and compliance to follow-up

In the intervention group, a total of 6.5% (95% CI: [6.0, 7.1%]; *n* = 455) were HPV–positive (Table 3). The HPV prevalence decreased with age from 7.3% (95% CI: [6.3, 8.4%]) at age 65 to 67 to 6.1% (95% CI: [5.4, 6.8%]; *p* = 0.05) at age 68 to 69. The HPV prevalence was higher in vaginal self-samples (8.0%, 95% CI: [6.9, 9.3%]) compared to clinician-collected cervical samples (5.9%, 95% CI: [5.2, 6.6%]; *p* < 0.001). Of those HPV–positive, HPV16/18 was found in 25.5% (95% CI: [21.5, 29.7%]) and HPV other types in 74.5% (95% CI: [70.2, 78.4%]) (Table 3). Insufficiently screened women had higher HPV prevalence (11.5%, 95% CI: [8.7, 14.9%]) as compared to sufficiently screened women (6.2%, 95% CI: [5.6, 6.8%]; *p* < 0.001). Among the 161 women with an HPV–positive self-sample, 99.4% (95% CI: [96.6, 100%]; *n* = 160) completed follow-up testing at their GP within 180 days, with one woman attending follow-up after 180 days. Of those HPV–positive, 96.4% (*n* = 439/455) had a record of either cytology (*n* = 164) or/and histology (*n* = 275) follow-up during the study period. In the reference group, a total of 4.0% (95% CI: [2.7, 5.7%]; *n* = 30) were HPV–positive; of whom 46.7% (95% CI: [28.3, 65.7%]) had HPV16/18 detected (Table 3). Among the HPV–positive; 96.7% (*n* = 29/30) had a record of either cytology (*n* = 7) or/and histology (*n* = 22) follow-up during the study period.

### Histological outcomes

Of the 6,965 HPV-tested women in the intervention group; 357 women (5.1%) had histology results registered during follow-up, 275 among HPV–positive and 82 among HPV–negative (Table 4). Of those with histology, 44 (12.3%, 95% CI: [9.1, 16.2%]; *n* = 44/357) had CIN2+ diagnosed (12 CIN2, 22 CIN3, 6 CIN, and 4 cancers) (Table 4). In the intervention group, the percentage of CIN2+ lesions diagnosed were higher in insufficiently screened (1.4%, 95% CI: [0.5, 2.9%]; *p* = 0.05; *n* = 6/443) but not statistically different from the percentage diagnosed among sufficiently screened (0.6%, 95% CI: [0.4, 0.8%]; *n* = 38/6,522). Among the 743 tested women in the reference group; 75 women (10.1%) had histology results registered and 11 (14.6%, 95% CI: [7.6, 24.7%]; *n* = 11/75) had CIN2+ diagnosed (3 CIN2, 3 CIN3, 1 CIN, and 4 cancers). The number of CIN3+ lesions diagnosed was significantly higher in the intervention group (2.3 per 1,000 eligible women; 95% CI: [1.5, 3.4]; *p* < 0.001) as compared to the reference group (0.2 per 1,000 eligible women; 95% CI: [0.1, 0.4]) (Table 4). An even more pronounced tendency was seen for CIN2+ lesions in the intervention group (3.9 per 1,000 eligible women, 95% CI: [2.9, 5.3]; *p* < 0.001) as compared to the reference group (0.3 per 1,000 eligible women, 95% CI: [0.2, 0.6]). For the harm–benefit ratio, 11.6 (95% CI: [8.5, 15.8]; *p* = 0.69) and 19.6 (95% CI: [13.2, 29.2]; *p* = 0.62) colposcopies were performed to detect one CIN2+ and CIN3+, respectively, in the intervention group as compared to 10.1 (95% CI: [5.4, 18.8]) and 15.8 (95% CI: [7.4, 34.0]) colposcopies in the reference group (Table 4).

**Table 4:** Histological outcomes and number of colposcopies performed per CIN2+/CIN3+ case detected in the intervention and reference groups.

	Intervention group		Reference group	
**Eligible women**	11,192		33,387	
**Tested women**	6,965		743	
Women with adequate histology*	357		75	
**Worst histology result**				
<CIN2	313		64	
CIN2+**	44		11	
CIN3+***	26		7	
Cervical cancer	4		4	
**CIN2+ cases according to age**		*p*-value^¤^		*p*-value^¤^
65–67 (%, [95% CI])	12 (0.5, [0.2, 0.8])	0.30	5 (1.6, [0.5, 3.7])	0.82
68–69 (%, [95% CI])	32 (0.7, [0.5, 1.0])		6 (1.4, [0.5, 3.0])	
**CIN2+ cases according to screening history at age 50–64**				
Insufficiently screened *n* (%, [95% CI])	6 (1.4, [0.5, 2.9])	*p* = 0.05	3 (4.6, [0.9, 12.9])	*p* = 0.03
Sufficiently screened *n* (%, [95% CI])	38 (0.6,[0.4, 0.8])		8 (1.2, [0.5, 2.3])	
**Histology results per 1,000 eligible women**^**a**^ [95% CI]				*p*-value^¤¤^
<CIN2	28.0 [25.0, 31.1]		1.9 [1.5, 2.5]	*p* < 0.001
CIN2+	3.9 [2.9, 5.3]		0.3 [0.2, 0.6]	*p* < 0.001
CIN3+	2.3 [1.5, 3.4]		0.2 [0.1, 0.4]	*p* < 0.001
Cervical cancer	0.4 [0.09, 0.9]		0.1 [0.03, 0.3]	*p* = 0.10
**Colposcopies**				
Number of colposcopies performed	511^##^		111^##^	
Number of colposcopies performed per CIN2+ case [95% CI]	11.6 [8.5, 15.8]		10.1 [5.4, 18.8]	0.69
Number of colposcopies performed per CIN3+ case [95% CI]	19.6 [13.2,29.2]		15.8 [7.4, 34.0]	0.62

CI, confidence interval. Adequate: women with inadequate or missing histology results were excluded.

* From biopsy, ECC or LEETZ from the worst diagnosis identified during follow-up.

** CIN2+ includes: CIN2, CIN3/AIS, CIN and cancer.

*** CIN3+ includes: CIN3/AIS and cancer.

^a^Unadjusted for age and screening history at age 50–64.

^¤^*p*-Value: comparison within group.

^¤¤^*p*-Value: comparison between intervention and reference group.

^##^Some women had >1 biopsy during follow-up and thus the number of colposcopies performed were higher than the number of women with adequate histology.

Among the 455 HPV–positive women in the intervention group, 275 women (60.4%) had histology results registered during follow-up. Of those, 43 had CIN2+ detected (15.6%, 95% CI: [11.6, 20.5%]; *n* = 43/275). The percentage of CIN2+ lesions diagnosed among HPV16/18 positive with histology (17.2%, 95% CI: [10.3, 26.1%]; *p* = 0.60; *n* = 17/99) was higher but not statistically different from the percentage diagnosed among HPV other type positive (14.8%, 95% CI: [9.9, 20.9%]; *n* = 26/176). Among the 82 HPV–negative women (all with clinician-collected cervical samples) with histology fewer than three cancers were detected.

### Discussion

Offering a catch-up HPV test to 65- to 69-year-old women, including the possibility to allow women to choose between a clinician-collected sample or vaginal self-sampling, resulted in an overall uptake of 62.2% and an HPV prevalence of 6.5%. The intervention was associated with a significantly higher CIN2+ detection rate as compared to the reference group (3.9 per versus 0.3 per 1,000 eligible women) and was not associated with a significantly higher number of colposcopies needed to detect one CIN2+ (11.6 versus 10.1, respectively). Whereas clinician-based sampling was the preferred screening modality overall (71.1%), a substantially higher percentage of insufficiently screened women underwent self-sampling than sufficiently screened women (52.1% versus 27.3%).

The screening uptake of 62.2% in the intervention group was substantially higher than the uptakes of 44.0% [37] and 41% to 43.3% [17,38] reported by Danish and Swedish studies targeting women aged 69 and above. The most likely explanations for the improved uptake were the lower age in our study population, providing women the choice between two screening modalities, and using reminders. Although clinician-based sampling was the preferred screening modality, the fact that almost every third woman tested (28.9%) selected self-sampling confirms that providing women the choice is essential and could increase uptake beyond offering clinician-based sampling alone [39]. If follow-up to the cytology test at the GP is poor among HPV–positive self-samplers, the benefit of offering self-sampling could be compromised [20]. In this study, the compliance to follow-up was remarkably high (99%) and was achieved without using an intensive follow-up protocol as used in other studies [20]. Although insufficiently screened women constitute a minority of the female population [10], nevertheless 23% in this study, they are at high risk of cervical cancer and, thus, of particular importance to reach [10,11]. Encouragingly, we observed that the screening uptake by an opt-in self-sampling strategy was almost 2 times higher among insufficiently screened women than among sufficiently screened women (52.1% versus 27.3%). Yet, vaginal self-sampling does not appeal to all women and data from the UK demonstrates that another promising screening modality could be to offer women the opportunity of non-speculum clinician-based sampling at the GP to overcome the pain associated with cervical sampling after menopause [39]. In the intervention group, we found a CIN2+ prevalence of 12.3% among tested women with histology follow-up. This was lower than reported in Danish women aged 69 and above with histology (18%) [16] and in Swedish women aged 56 to 60 with persistent HPV infections (23%) [40], but still higher than the very low CIN2+ prevalence of 0.2% to 1.0% among Swedish women aged 56 and above [41,42]. These differences were probably due to differences in screening strategy (cytology versus HPV) [13], screening history [10], age, and diagnostic strategies [22]. The true CIN2+ prevalence in postmenopausal women is difficult to estimate due to well-known diagnostic challenges resulting in increased risk of missing disease in the cervical canal [22]. A recent Danish study among older screen-positive women demonstrated that more than half of all CIN2+cases detected in the LLETZ specimens were missed by cervical biopsies, suggesting a significant risk of underdiagnosis [22]. Thus, the CIN2+ prevalence strongly depends on the diagnostic strategy and could be underestimated in our study. Ideally, eligible women in all Danish regions should have been individually randomized to the intervention and reference group instead of being allocated to the groups based on their geographical location. Unfortunately, this was not feasible from an organizational point of view [31]. Instead, we used the remaining four Danish regions without an organized HPV screening offer as reference group. For such a comparison to be valid, it is necessary to assume similar cervical cancer incidence rates across regions before the start of this study. This assumption was confirmed by NORDCAN data, showing comparable average age-standardized cervical cancer incidence rates across regions from 2007 to 2016 among women aged 65 and above [43]. Still, indications for cervical sampling in this age group likely differed between the groups. In the reference group, cervical sampling was assumedly performed because of symptoms, e.g., postmenopausal bleeding and thus more severe disease situation which were supported by 36.4% of all CIN2+ cases were cancer as compared to 9.1% in the intervention group. In comparison, women in the intervention group were more likely to be asymptomatic and thus at lower cancer risk. Therefore, comparison with the reference group should be done with caution. Part of the differences in the CIN2+ detection between groups could also be explained by variances in the distribution of age and screening history. Yet, we accounted for this by stratifying the study outcomes for these potential confounders.

In all screening initiatives, it is essential to carefully evaluate the trade-off between benefits and harms. From a population-based perspective, the intervention contributed to a significantly higher detection of CIN2+ and CIN3+ per 1,000 eligible women. Not all CIN3 lesions, and especially CIN2, detected at age 65 and older would have progressed into cancer in the remaining lifespan [7]; thus, the detection and treatment of these precancer lesions could be considered as overtreatment. However, the degree to which CIN2/CIN3 lesions that will progress or spontaneously regress has not been studied in older women [44]. According to Bekos and colleagues [45], the woman’s age has a substantial impact on the natural history of CIN, independent of CIN grade and hrHPV infection. To detect one CIN2+ case in the intervention and reference groups, comparable numbers of colposcopies were required (11.6 versus 10.1, respectively). Still, the estimate of 11.6 in the intervention group was significantly higher than the 5.4 colposcopies (defined by histological samples using LEETZ or biopsy) per CIN2+ case reported in the Danish HPV catch-up study targeting women aged 69 and above [16], suggesting that benefit–harm ratio was less favorable for the younger age group in our study. The amount of potential overdiagnosis was also considerable, as per 1,000 eligible women, 28.0 women had colposcopy performed to exclude the presence of CIN2+ as compared to 1.9 in the reference group. The high number of unnecessary colposcopies was likely influenced by our sensitive and conservative screening triage algorithm; we referred all women with HPV16/18 directly to colposcopy, and at the 12-month repeat screening, we performed co-testing and referred all women with HPV (regardless of genotype) or ASC-US and worse to colposcopy. Direct colposcopy referral of HPV16/18 screen-positive women seemed sensible, as 17% had CIN2+ detected which was within the commonly accepted risk-threshold (10% to 20%) for colposcopy referral in Europe [46]. Still, modification of the triage algorithms is warranted to reduce the number of unnecessary colposcopies and women placed in surveillance cycles of unclear end. In ongoing studies [47], the performance and referral rates of different triage strategies using p16/Ki67 dual stain cytology [48], extended genotyping [49], and DNA host cell methylation [50] are being evaluated.

The key strength of the study was that the intervention was undertaken within a real-world setting of a national screening program, using the same screening invitation protocol and follow-up algorithms that are used routinely in the Danish program. Furthermore, this study included several times as many women as the previous studies exploring the utility of vaginal self-sampling among older women [38,41,44]. The use of individual-level screening data from the nationwide DPDB [28] ensured completeness of the study outcomes and reduced risk of selection and information bias. As 96.4% of the intervention group and 96.7% of the reference group had either cytology or/and histology follow-up after an HPV–positive test result, the impact of loss to follow-up within the study period on the detection of CIN2+/CIN3+ can be expected to be limited. The high compliance to histology follow-up among test-positive women reduced the risk of underestimating the number of colposcopies performed; yet, there was a risk of underestimation as no procedure code exists for colposcopy in the DPDB [28]. Hence, women may have had a colposcopy performed without having histology material sampled. However, the magnitude of this would be expected to be low as national guidelines state that that all women referred for colposcopy should have a minimum of 4 biopsies collected [35]. Although, the study outcomes were stratified by age and screening history, residual confounding from smoking and sexual behavior remain a point of concern. However, the impact of smoking on the detection of CIN2+/CIN3+ was considered limited as one study found no association between screening at older age and risk of cervical cancer when adjusting for smoking [51]. Besides, survey data from younger women [52] found that sexual behavior was only slightly associated with cervical cancer screening participation. Thus, we find it unlikely that this factor substantially confounded the results in this older population.

Our study informs the current debate as to whether women over 65 years of age should be offered a catch-up HPV test if they never had an HPV test. Offering catch-up HPV screening resulted in higher detection of CIN2+ and CIN3+ without significantly increase in the number of colposcopies per detected CIN2+/CIN3+. Furthermore, women with HPV negative screening results in this study will be expected to have an extremely low risk of developing cervical cancer in their remaining life time [14]. Thus, the intervention might be anticipated to yield a greater cervical cancer prevention than no HPV screening intervention. On the other hand, even with the potential risk of underestimation, the rather low CIN2+ prevalence (12.3%) together with the considerable amount of potential overdiagnosis and recognized clinical management issues justify that the choice of the future screening strategy for this older age group should be based on the availability of resources and attitudes to cervical cancer risk in each country [16,22]. As previous screening participation and screening results are key risk markers for cervical cancer at older ages [10,11], one could argue that a satisfactory cost-benefit balance of catch-up HPV interventions would be to only target women aged 65 and above if they have been insufficiently screened instead of targeting all sufficiently screened women who exited the program with normal cytology but never had an HPV test. This differentiated strategy was supported by our intervention data showing significantly higher HPV prevalence as well as a tendency towards higher CIN2+ detection in insufficiently screened as compared to sufficiently screened women. Yet, this differentiated strategy requires up-to-date screening registries, an option not in place everywhere.

In countries with limited resources, collecting an opportunistic cervical cytology sample due to gynecological symptoms seems appropriate from a clinical point of view based on the higher CIN2+ detection per 1,000 tested women in the reference as compared to the intervention group (14.8 versus 6.3 per 1,000 tested, reflecting a per protocol analysis).

Based on this study, vaginal self-sampling might be the optimal screening modality for women aged 65 and above based on its ability to reach older insufficiently screened women and its more favorable cost than clinician-based sampling [21]. Our results may be generalized to countries with comparable organization of the cervical cancer screening program with free-of-charge screening and access to follow-up testing; however, the effect size may depend on the screening history in the included birth cohorts, screening uptake, compliance to follow-up, and especially the diagnostic management of the screen-positive women [22] in each country.

## Conclusions

The HPV catch-up screening intervention was associated with higher CIN2+ detection as compared to no screening intervention but longer follow-up is necessary to observe if the intervention translates into fewer cervical cancers and deaths in the screened women. Vaginal self-sampling constituted a valuable tool to identify older insufficiently screened women at risk of cervical cancer. Whether screening strategies for women aged 65 and above who have never had an HPV test should be provided to all individuals or differentiated depending on previous screening history may depend on the availability of resources in each country, including a careful evaluation of the trade-off between benefits and harms.

## Supporting information

S1 Text Trend Statement Checklist. (DOCX)

S2 Text Codes for hysterectomy and cervical amputation. (DOCX)

## Abbreviations

CDR: Central Denmark Region
CI: confidence interval
CIN: cervical intraepithelial neoplasia
CPR: Civil Personal Registration
DPDB: Danish Pathology Data Bank
ECC: endocervical curettage
GP: general practitioner
HPV: human papillomavirus
LLETZ: large loop excision of the transformation zone
