# Supplementary figures and images for: Value of a catch-up HPV test in women aged 65 and above: A Danish population-based nonrandomized intervention study
Source: PLoS Med. 2023 Jul 6;20(7):e1004253. doi: 10.1371/journal.pmed.1004253 (PMC10325045; doi:10.1371/journal.pmed.1004253)

**S1 Trend Statement Checklist**


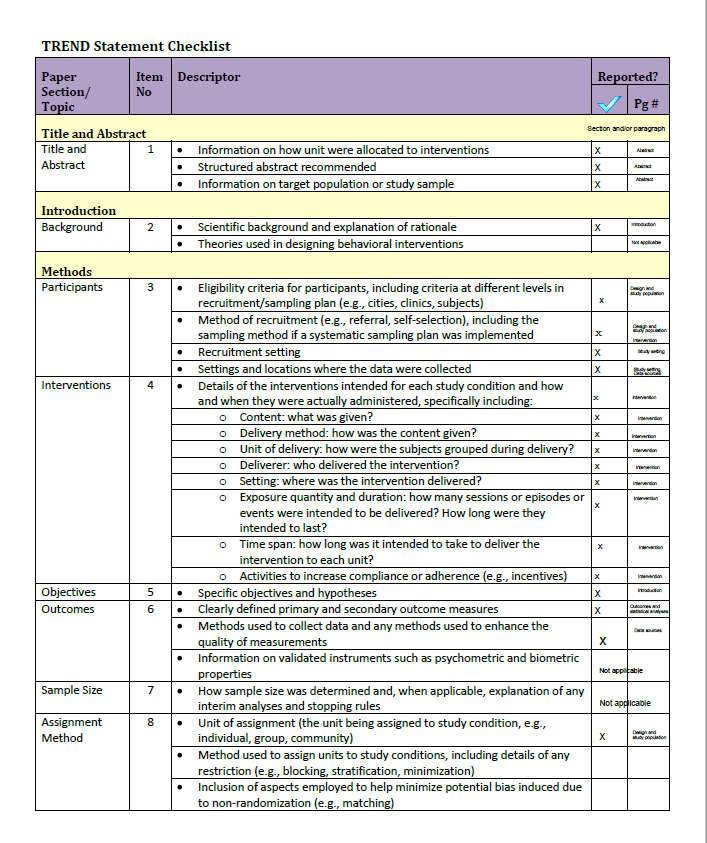


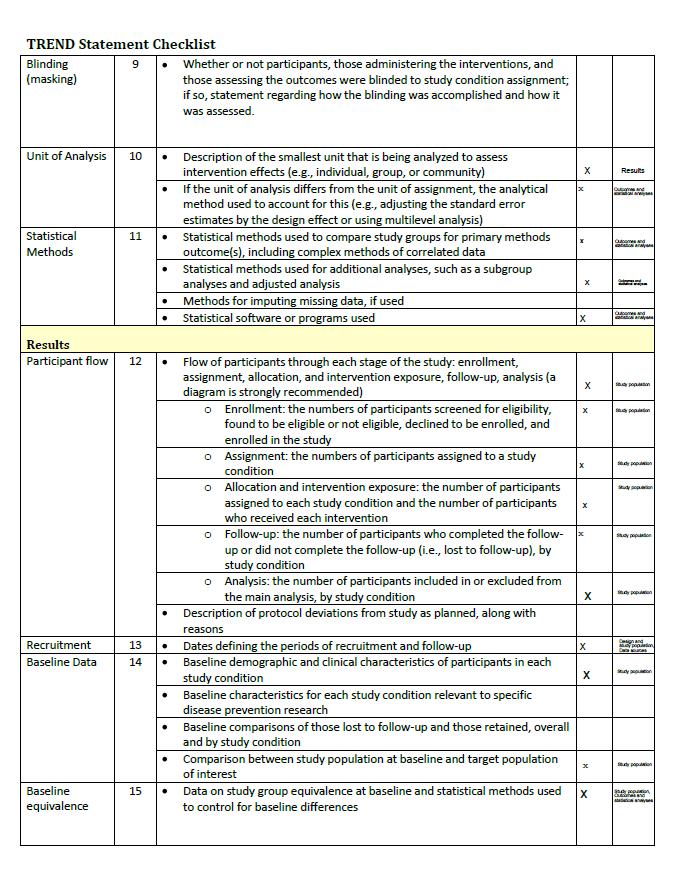


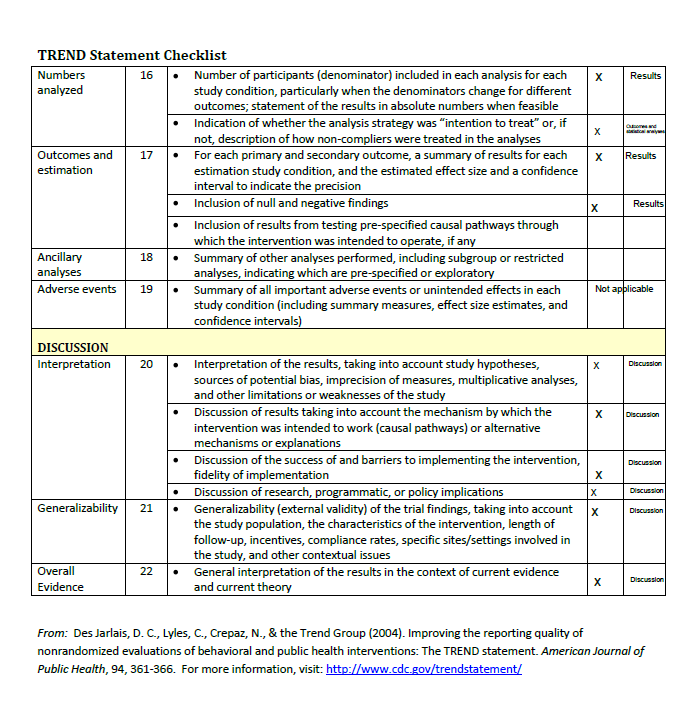

Supplement: S1 Text — (DOCX) [file pmed.1004253.s001.docx]
