# Supplementary material for: Value of a catch-up HPV test in women aged 65 and above: A Danish population-based nonrandomized intervention study
Source: PLoS Med. 2023 Jul 6;20(7):e1004253. doi: 10.1371/journal.pmed.1004253 (PMC10325045; doi:10.1371/journal.pmed.1004253)
Supplement: S2 Text — (DOCX) [file pmed.1004253.s002.docx]

**S2 Codes for hysterectomy and cervical amputation
A complete list of ICD-8 and ICD-10 codes used to identify women with hysterectomy and cervical amputation.**

The Danish National Patient Registry was searched for the following surgical procedures: abdominal hysterectomy (KLCD00), laparoscopic assisted hysterectomy(KLCD11), laparoscopic hysterectomy (KLCD01, KLCD04, KLCD97), vaginal hysterectomy (KLCD10, KLEF13), radical hysterectomy (KLCD30, KLCD31, KLCD40), cesarean section and total hysterectomy (KMCA33), other hysterectomy (KLCD96), trachelectomy (KLDC20, KLDC23), and amputation of the cervix (KLEF00B, KLDC10, KLDC13, KLDC96). Supravaginal hysterectomy KLCC10, KLCC11, KLCC20).
